# A joint cross-border investigation of a cluster of multidrug-resistant tuberculosis in Austria, Romania and Germany in 2014 using classic, genotyping and whole genome sequencing methods: lessons learnt

**DOI:** 10.2807/1560-7917.ES.2017.22.2.30439

**Published:** 2017-01-12

**Authors:** Lena Fiebig, Thomas A Kohl, Odette Popovici, Margarita Mühlenfeld, Alexander Indra, Daniela Homorodean, Domnica Chiotan, Elvira Richter, Sabine Rüsch-Gerdes, Beatrix Schmidgruber, Patrick Beckert, Barbara Hauer, Stefan Niemann, Franz Allerberger, Walter Haas

**Affiliations:** 1 Respiratory Infections Unit, Department for Infectious Disease Epidemiology, Robert Koch Institute, Berlin, Germany; 2 These authors contributed equally to this work; 3 Molecular and Experimental Mycobacteriology, Research Center Borstel, Leibniz-Center for Medicine and Biosciences, Borstel, Germany; 4 National Institute of Public Health – National Center for Communicable Diseases Surveillance and Control, Bucharest, Romania; 5 Local Public Health Office Ingolstadt, Ingolstadt, Germany; 6 Austrian Reference Laboratory for Mycobacteria, Austrian Agency for Health and Food Safety (AGES), Vienna, Austria; 7 Clinical Hospital of Pneumology, Tuberculosis National Reference Laboratory, Cluj-Napoca, Romania; 8 Pneumology Institute “Marius Nasta”, Bucharest, Romania; 9 Labor Dr. Limbach, TB Laboratory, Heidelberg, Germany; 10 National Reference Center (NRC) for Mycobacteria, Research Center Borstel, Borstel, Germany; 11 Tuberculosis Patient Service, Health Service of the City of Vienna, Vienna, Austria; 12 German Center for Infection Research, Partner Site Hamburg-Borstel-Lübeck, Borstel, Germany

**Keywords:** tuberculosis, multidrug-resistant, molecular epidemiology, sequence analysis, genotyping, contact tracing, transmission, Europe, human migration

## Abstract

Molecular surveillance of multidrug-resistant tuberculosis (MDR-TB) using 24-loci MIRU-VNTR in the European Union suggests the occurrence of international transmission. In early 2014, Austria detected a molecular MDR-TB cluster of five isolates. Links to Romania and Germany prompted the three countries to investigate possible cross-border MDR-TB transmission jointly. We searched genotyping databases, genotyped additional isolates from Romania, used whole genome sequencing (WGS) to infer putative transmission links, and investigated pairwise epidemiological links and patient mobility. Ten isolates from 10 patients shared the same 24-loci MIRU-VNTR pattern. Within this cluster, WGS defined two subgroups of four patients each. The first comprised an MDR-TB patient from Romania who had sought medical care in Austria and two patients from Austria. The second comprised patients, two of them epidemiologically linked, who lived in three different countries but had the same city of provenance in Romania. Our findings strongly suggested that the two cases in Austrian citizens resulted from a newly introduced MDR-TB strain, followed by domestic transmission. For the other cases, transmission probably occurred in the same city of provenance. To prevent further MDR-TB transmission, we need to ensure universal access to early and adequate therapy and collaborate closely in tuberculosis care beyond administrative borders.

## Background

Tuberculosis (TB) and its multi- and extensively drug-resistant forms (M/XDR-TB) are a major global public health concern. The World Health Organization (WHO) estimates that 9.6 million people worldwide fell ill with TB in 2014, of those ca 480,000 cases with MDR-TB [1]. Where second-line drug susceptibility testing (DST) is available, (pre)XDR-TB is frequently detected [2,3]. These patients have a high risk of death [3].

To control this infectious disease, it is key to understand and interrupt the spread of TB and M/XDR-TB. TB transmission can be traced by classic and by molecular epidemiological methods. Classic methods include contact and source case investigations based on patient interviews. Molecular methods examine the genetic relationship between the isolates of the *Mycobacterium tuberculosis* complex. Common genotyping methods include spacer oligonucleotide typing (spoligotyping) and 24-loci mycobacterial interspersed repetitive units variable number of tandem repeats (24-loci MIRU-VNTR) analysis, both targeting specific small parts of the genome. Whole genome sequencing (WGS) queries the entire mycobacterial genomic material. It has higher discriminatory power and may indicate the directionality and sequence of transmission events [4-7]. Moreover, WGS permits identification of genes and mutations that mediate drug resistance [8-11]. WGS has been employed to analyse and review TB outbreaks in different settings [5,12,13]. Recently, it has become increasingly affordable and routinely applicable [8,14,15].

Austria, Romania and Germany are European Union (EU) Member States with, respectively, TB notification rates of 6.8, 79.7 and 5.6 cases per 100,000 population, rather similar proportions of MDR-TB among new laboratory-confirmed TB cases with DST results of 4.8%, 6.4% and 3.1%, yet very different absolute numbers of detected MDR-TB cases with 20, 517 and 87 cases in 2014 [2].

None of the three countries has an area-wide integrated molecular surveillance for TB as established in the Netherlands [16], the United Kingdom (UK) [17] or the United States (US) [18]. However, the National Reference Laboratories (NRLs) for Mycobacteria in Austria and Germany systematically type M/XDR-TB isolates. Germany submits the results to the genotyping database of the European Centre for Disease Prevention and Control (ECDC) [19].

In March 2014, the Austrian NRL at the Austrian Agency for Health and Food Safety (AGES) detected a molecular cluster of five MDR-TB cases. The question arose whether MDR-TB transmission had occurred within Austria, which had never been observed before. Links to Romania and Germany prompted the three countries to investigate the MDR-TB cluster jointly within given legal contexts and with unchanged in-country responsibilities, with the aim of tracing the MDR-TB transmission.

## Methods

### Collaboration

The investigation team consisted of the national TB contact points for WHO and ECDC or representatives acting on their behalf, the NRLs and the responsible local public health authorities in Austria, Romania and Germany. Collaboration was maintained by monthly telephone conferences from April to October 2014.

### Case inclusion

Cases were included without restriction in time when the isolate, collected in any of the three countries and recorded in any typing databases by the NRLs, shared the same spoligotype and 24-loci MIRU-VNTR pattern as in the initial cluster detected in Austria in March 2014. Five MDR-TB cases from one administrative district in Romania were included based on epidemiological information in the absence of molecular typing data. No epidemiological links pointing to other districts in Romania were identified.

### Drug susceptibility testing

Isolates were gained by culturing specimens in liquid (BACTEC MGIT 960, Becton Dickinson Diagnostic Systems, Sparks, US) and on solid Löwenstein-Jensen (LJ) media.

In Austria and Germany, DST was done using the Mycobacteria Growth Indicator Tube (MGIT) system with BACTEC MGIT 960 growth supplement for DST in the MGIT 960 instrument (Becton Dickinson Diagnostic Systems, Sparks, MD). For cycloserine, the proportion method employed was modified according to Canetti [20]. In Romania, specimens were cultured on LJ medium. The proportion method was used to test isoniazid, rifampicin, ethambutol, streptomycin, kanamycin, amikacin, capreomycin, ofloxacin and ethionamide.

### Genotyping

On extracted genomic DNA from the mycobacterial strains, spoligotyping and 24-loci MIRU-VNTR was done following standard protocols [21,22].

### Whole genome sequencing and sequence data analysis

Libraries for sequencing were prepared from extracted genomic DNA with the Nextera XT library preparation kit and sequenced on the Illumina MiSeq next generation sequencing (NGS) platform in a 2 × 301 bp paired-end run (Illumina, San Diego, US).

WGS data of sequenced isolates were submitted to the EMBL-EBI ENA sequence read archive (accession number: ERP013444). Resulting reads were mapped to the *M. tuberculosis* H37Rv genome (GenBank accession number: NC_000962.3) with the SARUMAN exact alignment tool [23]. The mean genomic coverage was at least 45-fold, with more than 99% of the reference genome covered for all isolates. Variants were called from mapped reads by in-house Perl scripts, asking for a minimum coverage of 10 reads and a minimum allele frequency of 75% as detection thresholds. Combining detected single nucleotide polymorphisms (SNPs) of all isolates, positions that matched the threshold levels in at least 95% of all isolates were considered as valid and used for a concatenated sequence alignment excluding variants in resistance-associated or repetitive regions of the genome.

We employed the BioNumerics software (Applied Maths NV, Belgium) to build a neighbour-joining tree from the 708 concatenated SNP positions. Putative transmission groups were predicted with a cut-off of 12 distinct SNP positions (referred to as WGS_12SNPs_ clusters) [24].

All variants located on genes that were previously associated with mutations conferring drug resistance were extracted from the full set of detected variants, and the derived subset of variants was manually annotated with published data [8,25-30].

WGS was performed at the NRL at the Research Center Borstel in Germany.

### Epidemiological investigation

We used a self-designed form in all three countries to systematically compile patient information, direct epidemiological links (exposure of at least 8 hours or at least 40 hours to, respectively, a sputum smear- or culture-positive but sputum smear-negative source case) [31,32], and spatio-temporal information in terms of the patients’ city and country of stay per month from January 2009 to July 2014. The data sources were records of the responsible authorities and re-interviews of the patients III, IV, V, VI. The others could not be contacted, had reportedly moved away or did not follow the invitation by the authorities.

We compiled these data into a line list using Microsoft Excel and analysed them descriptively.

### Legal framework and data protection

Patient data had been collected as part of routine case notification and contact investigation according to the Tuberculosis Law (Tuberkulosegesetz) in Austria, Law Number 95/2006 on Health Reform in Romania, and the Protection against Infection Act (Infektionsschutzgesetz; IfSG) in Germany.

The collection of direct person-to-person links required international sharing of all patients’ names. The Decision Number 1082/2013/EU of the European Parliament and of the Council [33] stipulates that proper authorities may communicate personal data for contact tracing purposes through selective exchanges in the European Early Warning and Response System (EWRS). In Germany, authorisation to collect personal data under the terms of section 16(1) IfSG lies with local public health authorities while the national authority’s administrative involvement in handling personal data (section 25(1), IfSG) is restricted to international travellers (section 12(7) International Health Regulation Implementation Act).

Accordingly, in Germany, one of the responsible local authorities compiled the patients’ names, assigned random unique identifiers (IDs) and redistributed the key to authorities in charge of the patients in the three countries. The form was completed using the ID, the key destroyed and anonymous data shared with the German national TB contact point at the Robert Koch Institute (RKI) for analysis.

The investigation protocol had been positively evaluated by data protection and legal departments of the RKI.

## Results

### Austria

**Figure 1:**
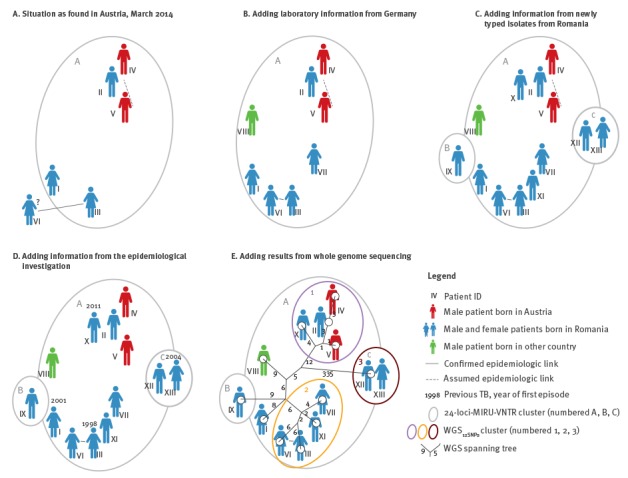
Cluster of multidrug-resistant tuberculosis in Austria, Romania and Germany, 2010 to 2014 (n = 13)

ID: unique patient identifier; TB: tuberculosis; WGS: whole genome sequencing.

In March 2014, *M. tuberculosis* (non-Beijing genotype) isolates from five MDR-TB patients in Austria were found to share the same spoligotype and 24-loci MIRU-VNTR pattern ‘A’. Three patients (I–III) diagnosed from 2010 to 2012, originated from the same city in Romania (Figure 1A, Table 1). They had moved to two different cities in Austria, seeking medical care for their complicated MDR-TB. Two patients (IV and V) had been diagnosed with new MDR-TB in June 2013. They were residents of the same Austrian city to which patients I and II had moved and had no history of migration or international travel.

The panels present the findings of our cluster investigation in chronological order.

**Table 1:** Cluster of multidrug-resistant tuberculosis in Austria, Romania and Germany, demographic and clinical characteristics of the investigated patients, 2010 to 2014 (n = 13)

Patient ID	Country of residence at the beginning of the investigation	Sex	Age group (years)	Country of birth	Month and year of diagnosis of current episode	Previous TB (year of diagnosis)	Site of disease
**I**	Austria	Female	30–39	Romania	03/2010	Yes (2001)	Pulmonary
**II**	Austria	Male	50–59	Romania	01/2011	No	Pulmonary
**III**	Austria	Female	30–39	Romania	03/2012	Yes (1998, 2003)	Pulmonary
**IV**	Austria	Male	40–49	Austria	06/2013	No	Pulmonary
**V**	Austria	Male	50–59	Austria	06/2013	No	Pulmonary
**VI**	Germany	Female	30–39	Romania	12/2011	No	Pulmonary
**VII**	Germany	Female	30–39	Romania	05/2011	No	Pulmonary
**VIII**	Germany	Male	30–39	Nigeria	07/2011	No	Extrapulmonary
**IX**	Romania	Male	40–49	Romania	01/2004	No	Pulmonary
**X**	Romania	Male	50–59	Romania	12/2011	Yes (2011)	Pulmonary
**XI**	Romania	Male	30–39	Romania	01/2014	No	Pulmonary
**XII**	Romania	Male	20–29	Romania	12/2013	No	Pulmonary
**XIII**	Romania	Female	60–69	Romania	01/2014	Yes (2004)	Pulmonary

ID: unique patient identifier; TB: tuberculosis.

Contact tracing did not confirm any epidemiological link between patients I to IV. However, a link between patients IV and V was assumed; they had both frequented the vicinity of the railway station and had problematic alcohol use.

Patient III reported having a sister diagnosed with MDR-TB living in Germany. This prompted the AGES to share the spoligotype and MIRU-VNTR pattern (Table 2) with Germany.

**Table 2:** Cluster of multidrug-resistant tuberculosis in Austria, Romania and Germany, bacteriological confirmation and spoligotype and 24-loci MIRU-VNTR pattern of the isolates from the investigated patients, 2010 to 2014 (n = 13)

ID	Bacterial confirmation	Archive run accession	Spoligotype	24-loci MIRU-VNTR																							
				154	424	577	580	802	960	1644	1955	2059	2163b	2165	2347	2401	2461	2531	2687	2996	3007	3171	3192	3690	4052	4156	4348
I	ND	ERR1163047	1111111100111111111111111111111100001111111	2	2	3	2	2	3	4	2	2	4	2	4	2	2	5	1	5	3	3	2	3	5	2	2
II	Culture-pos, NAAT-pos, ssm-pos	ERR1163048	1111111100111111111111111111111100001111111	2	2	3	2	2	3	4	2	2	4	2	4	2	2	5	1	5	3	3	2	3	5	2	2
III	Culture-pos, NAAT-pos, ssm-pos	ERR1163049	1111111100111111111111111111111100001111111	2	2	3	2	2	3	4	2	2	4	2	4	2	2	5	1	5	3	3	2	3	5	2	2
IV	Culture-pos, NAAT-pos	ERR1163050	1111111100111111111111111111111100001111111	2	2	3	2	2	3	4	2	2	4	2	4	2	2	5	1	5	3	3	2	3	5	2	2
V	Culture-pos, NAAT-pos	ERR1163051	1111111100111111111111111111111100001111111	2	2	3	2	2	3	4	2	2	4	2	4	2	2	5	1	5	3	3	2	3	5	2	2
VI	Culture-pos, NAAT-pos, ssm-pos	ERR1163052	1111111100111111111111111111111100001111111	2	2	3	2	2	3	4	2	2	4	2	4	2	2	5	1	5	3	3	2	3	5	2	2
VII	Culture-pos, NAAT-pos, ssm-pos	ERR1163053	1111111100111111111111111111111100001111111	2	2	3	2	2	3	4	2	2	4	2	4	2	2	5	1	5	3	3	2	3	5	2	2
VIII	Microscopy of EP specimen-pos	ERR1163054	1111111100111111111111111111111100001111111	2	2	3	2	2	3	4	2	2	4	2	4	2	2	5	1	5	3	3	2	3	5	2	2
IX	Culture-pos	ERR1163055	1111111100111111111111111111111100001111111	2	2	3	2	3	3	3	2	2	4	2	4	2	2	5	1	5	3	3	2	3	5	2	2
X	Culture-pos, ssm-pos	ERR1163056	1111111100111111111111111111111100001111111	2	2	3	2	2	3	4	2	2	4	2	4	2	2	5	1	5	3	3	2	3	5	2	2
XI	Culture-pos, ssm-pos	ERR1163057	1111111100111111111111111111111100001111111	2	2	3	2	2	3	4	2	2	4	2	4	2	2	5	1	5	3	3	2	3	5	2	2
XII	Culture-pos, ssm-pos	ERR1163058	1111111111111111111111111111111100001111111	2	3	4	2	4	3	1	2	2	3	2	3	2	3	6	1	5	3	3	2	2	5	2	2
XIII	Culture-pos, ssm-pos	ERR1163059	1111111111111111111111111111111100001111111	2	3	4	2	4	3	1	2	2	3	2	3	2	3	6	1	5	3	3	2	2	5	2	2

ID: unique patient identifier; MIRU-VNTR ND: 24-loci mycobacterial interspersed repetitive units variable number of tandem repeats; no data; ssm: sputum smear microscopy; shaded cells: distinct molecular typing patterns.

### Germany

In early April, the NRL in Germany identified three isolates with MIRU-VNTR pattern ‘A’. One isolate referred to the sister of patient III (patient VI), the second to another woman born in Romania (patient VII), and the third to a man born in West Africa with extrapulmonary non-MDR-TB (patient VIII; Figure 1B).

As five patients (I–III, VI and VII) reportedly originated from the same city in Romania, the Romanian national TB contact point was informed. In mid-April 2014, all three countries held their first telephone conference and agreed upon a joint investigation.

### Romania

In Romania, in the absence of systematic MIRU-VNTR typing of MDR-TB strains, isolates from all five MDR-TB patients (IX–XIII) ever reported in the corresponding district were typed at the Austrian NRL. The isolate from patient IX had a unique MIRU-VNTR pattern ‘B’, the isolates from patients X and XI shared pattern ‘A’, and the ones from patients XII and XIII shared a distinct pattern ‘C’ and a different spoligotype (Figure 1C, Table 2).

### Epidemiological investigation

Investigation forms were completed for patients II–XIII by seven public health authorities by September 2014. For patient I, only a laboratory report was available.

All patients were adults, five women and eight men; six had experienced migration (I–III and VI–VIII). Nine had new TB, four (I, III, X and XIII) had had previous TB, the first TB diagnosis dating back to year 1998 (III). All but patient VIII had pulmonary TB (Table 1).

The two sisters (III and VI) were confirmed to have a direct epidemiological link between them. Direct links were ruled out for persons II, III, IV, V, VII, IX and XII, and unknown for VI, VIII, X, XI and XIII. The assumed link between cases IV and V was negated when re-interviewing the persons (Figure 1D).

**Figure 2:**
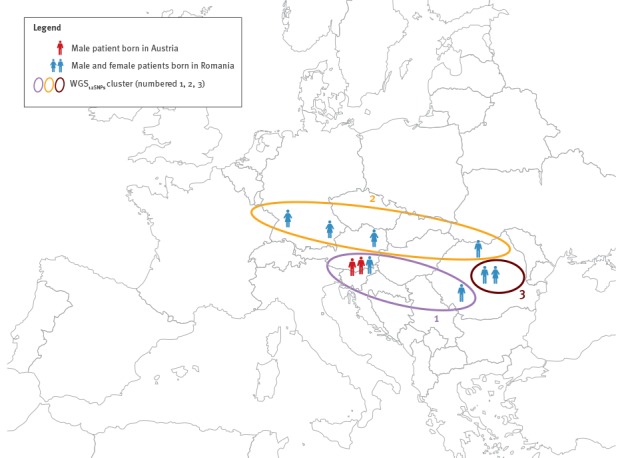
Geographical dimension of the three WGS_12SNPs_ cluster of multidrug-resistant tuberculosis, Austria, Romania and Germany, 2010 to 2014 (n = 10)

**Figure 3:**
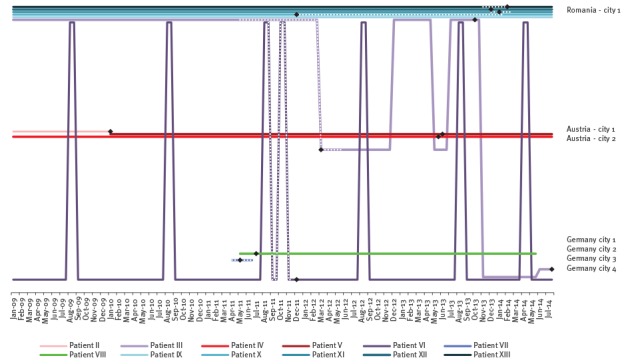
Patient mobility per city/country and month, cluster of multidrug-resistant tuberculosis, Austria, Romania and Germany, 2010 to 2014 (n = 10)

The two sisters (III and VI) had crossed borders presumably while being infectious (Figures 2 and 3). Other patients with migration background had moved before 2009 (II) or at an unknown date (I, VII, VIII). The mobility pattern did not preclude TB transmission events from patient II to patients IV and V in Austria, nor from patient III to patients X–XIII in Romania. The sisters III and VI had a space–time correlation in Romania in August 2011, however, only about one month before the beginning of the assumed infectious period of patient VI.

WGS: whole genome sequencing.

The persons are depicted in their country of residence at the beginning of the investigation, the colour refers to their country of birth (blue – Romania, red – Austria). The map background is used from RegioGraph (version 2015, GfK GeoMarketing GmbH, Bruchsal, Germany).

Dotted lines: assumed period of infectiousness; black diamonds: dates of notification for patients II–XIII.

### Whole genome sequencing

WGS was completed by August 2014. WGS_12SNPs_ divided cluster ‘A’ into two subgroups (one comprising patients II, IV, V and X, the other patients III, VI, VII and XI), and two separate cases (I and VIII). The third WGS_12SNPs_ cluster was congruent with genotyping pattern ‘C’ (Figure 1E). The isolates from patients II and IV, as well as II and V were distinct by 3 and 4 SNPs, respectively. Isolates from patients XII and XIII were genetically identical (0 SNPs). The isolates from the epidemiologically linked sisters were distinct by 12 SNPs.

The first two WGS_12SNPs_ clusters spanned across borders, while the third was domestic (Figure 2).

The detected mutations mediating resistance to first-line drugs correlated with phenotypic DST results. The isoniazid resistance-conferring mutation S315T in *katG* fully matched phenotypic isoniazid resistance; the same was observed for S450L or T400A in *rpoB* and rifampicin/rifabutin resistance and A146V in *pncA* and pyrazinamid resistance (information missing for patients IX–XIII). Two phenotypical ethambutol-susceptible isolates harboured the known resistance-mediating mutation M306I in *embB*; the resistant isolates showed either the mutation M306I or a combination of two mutations G406S and D1024N.

In addition, we detected resistance-mediating mutations for streptomycin (*rpsL* K43R) and kanamycin/amikacin (*rrs* 1401 A -> G). One of two quinolone-resistant isolates shows a mutation in *gyrA* (A288D), a quinolone resistance-associated gene. Among the five isolates phenotypically resistant to ethionamide, one harboured a frameshift insertion in *ethA.* Four out of eight phenotypical protionamide-resistant isolates, showed frameshift insertions in *ethA* (Tables 3 and 4). Patients in one WGS cluster shared a cluster-specific set of resistance-mediating mutations, patient X in cluster 1 and patient VI in cluster 2 had acquired an additional aminoglycoside resistance (*rrs* 1401 A -> G).

**Table 3:** Phenotypic drug susceptibility testing results, cluster investigation of multidrug-resistant tuberculosis, Austria, Romania and Germany, 2010 to 2014 (n = 13)

ID	H	R	Z	E	Eth	Pt	PAS	Rb	Cs	S	Amk	Kan	Cap	Ofl	Mox	Lev
I	Res	Res	Res	Sus	ND	Res	Sus	Res	Sus	Sus	Sus	ND	Sus	Res	Res	ND
II	Res	Res	Res	Res	ND	Res	Sus	Res	Sus	Res	Sus	ND	Sus	Sus	ND	ND
III	Res	Res	Res	Res	Res	Res	Sus	Res	Res	Res	Sus	ND	Sus	Res	Res	Res
IV	Res	Res	Res	Sus	ND	Res	Sus	Res	Sus	Res	Sus	ND	Sus	Sus	Sus	ND
V	Res	Res	Res	Sus	Res	Res	Sus	Res	Sus	Res	Sus	ND	Sus	Sus	Sus	ND
VI	Res	Res	Res	Res	Res	Res	Sus	Res	Sus	Res	Res	ND	Res	Sus	ND	ND
VII	Res	Res	Res	Res	Res	Res	Sus	Res	Sus	Res	Sus	ND	Sus	Sus	ND	ND
VIII	Res	Sus	Res	Sus	Res	Res	ND	ND	ND	Res	ND	ND	Sus	Sus	ND	ND
IX	Res	Res	ND	ND	ND	ND	ND	ND	ND	Res	ND	ND	ND	ND	ND	ND
X	Res	Res	ND	Res	Sus	ND	ND	ND	ND	Res	Res	Res	Res	Sus	ND	ND
XI	Res	Res	ND	Res	Sus	ND	ND	ND	ND	Res	Sus	Sus	Sus	Sus	ND	ND
XII	Res	Res	ND	Sus	Sus	ND	ND	ND	ND	Res	Sus	Sus	Sus	Sus	ND	ND
XIII	Res	Res	ND	Sus	Sus	ND	ND	ND	ND	Res	Sus	Sus	Sus	Sus	ND	ND

Amk: amikacin; Cap: capreomycin; E: ethambutol; Eth: ethionamide; ID: unique patient identifier; Kan: kanamycin; Lev: levofloxacin; Mox: moxifloxacin; ND: no data; H: isoniazid; Ofl: ofloxacin; PAS: para-aminosalicylic acid; Pt: protionamide; R: rifampicin; Rb: rifabutin; Res: resistant; Cs: cycloserine; S: streptomycin; Sus: susceptible; Z: pyrazinamide.

**Table 4:** Genotypic drug susceptibility testing results, cluster of multidrug-resistant tuberculosis, Austria, Romania and Germany, 2010 to 2014 (n = 13)

ID	H	R	R	Z	E	E	E	Eth/Pt	Eth/Pt	Ami	S	SM	PAS	FQ
	Rv1908c	Rv0667	Rv0667	Rv2043c	Rv3795	Rv3795	Rv3795	Rv3854c	Rv3854c	MTB000019	Rv3919c	Rv0682	Rv2764c	Rv0007
	katG [26]	rpoB [8,25]	rpoB [8,25]	pncA [27]	embB [8,28]	embB [8,28]	embB [8,28]	ethA [29]	ethA [29]	Rrs [8]	gidB [25]	rpsL [25]	thyA [30]	gyrA [8]
I	S315T ^a^	WT	S450L ^a^	A146V ^a^	WT	WT	D1024N ^b^	WT	Ins 802 ag ^b^	WT	Q125_ ^b^	WT	R222C^b^	A288D^b^
II	S315T ^a^	WT	S450L ^a^	A146V ^a^	M306I ^a^	WT	WT	Ins 1391 a ^b^	WT	WT	Q125_ ^b^	WT	WT	WT
III	S315T ^a^	WT	S450L ^a^	A146V ^a^	WT	G406S^a^	D1024N ^b^	WT	WT	WT	Q125_ ^b^	WT	WT	WT
IV	S315T ^a^	WT	S450L ^a^	A146V ^a^	M306I ^a^	WT	WT	Ins 1391 a ^b^	WT	WT	Q125_ ^b^	WT	WT	WT
V	S315T ^a^	WT	S450L ^a^	A146V ^a^	M306I ^a^	WT	WT	Ins 1391 a ^b^	WT	WT	Q125_ ^b^	WT	WT	WT
VI	S315T ^a^	WT	S450L ^a^	A146V ^a^	WT	G406S ^a^	D1024N ^b^	WT	WT	1401 A -> G ^a^	Q125_ ^b^	WT	WT	WT
VII	S315T ^a^	WT	S450L ^a^	A146V ^a^	WT	G406S ^a^	D1024N ^b^	WT	WT	WT	Q125_ ^b^	WT	WT	WT
VIII	S315T ^a^	WT	WT	A146V ^a^	WT	WT	WT	WT	WT	WT	Q125_ ^b^	WT	WT	WT
IX	S315T ^a^	WT	S450L ^a^	A146V ^a^	M306I ^a^	WT	D1024N ^b^	WT	WT	WT	Q125_ ^b^	WT	WT	WT
X	S315T ^a^	WT	S450L ^a^	A146V ^a^	M306I ^a^	WT	WT	Ins 1391 a ^b^	WT	1401 A- > G ^a^	Q125_ ^b^	WT	WT	WT
XI	S315T ^a^	WT	S450L ^a^	A146V ^a^	WT	G406S ^a^	D1024N ^b^	WT	WT	WT	Q125_ ^b^	WT	WT	WT
XII	S315T ^a^	T400A ^a^	S450L ^a^	WT	WT	WT	WT	WT	WT	WT	WT	K43R^1^	WT	WT
XIII	S315T ^a^	T400A ^a^	S450L ^a^	WT	WT	WT	WT	WT	WT	WT	WT	K43R^1^	WT	WT

Ami: aminoglycoside; E: ethambutol; Eth: ethionamide; FQ: fluroquinolones; ID: unique patient identifier; H: isoniazid; PAS: para-aminosalicylic acid; Pt: protionamide; R: rifampicin; S: streptomycin; Z: pyrazinamide; WT: wild type.

^a^ resistance mediating mutation.

^b^ resistance associated variant.

## Discussion

We investigated a molecular cluster of MDR-TB in Austria, Romania and Germany. WGS combined with epidemiological information showed that isolates from patient II, seeking medical care in Austria, differed from the subsequently diagnosed Austrian patients IV and V by only 3 and 4 SNPs, respectively. This suggested that two MDR-TB transmission events had occurred in Austria. Isolates from patients III, VI, VII and XI, who lived in three different countries but had the same city of provenance, differed by 6–12 SNPs from each other. Here, transmission is likely to have occurred before the patients moved abroad.

Close genetic similarity of isolates from different patients is highly unlikely to occur by chance. From well-described TB outbreaks we know that isolates gained within three years from patients with a direct epidemiological link usually differ by 5 or fewer SNPs [34,35]. In an outbreak of nine drug-susceptible TB cases in San Francisco, US, the isolates differed by 0–2 SNPs per any transmission event that had resulted in a secondary case [6]. In a similar investigation in Germany, differences of 0–3 SNPs were found (n = 31) [7]. From a retrospective study of TB outbreaks, Walker and colleagues derived that epidemiological linkage is expected to be consistent with sequenced isolates differing in up to 5 SNPs; the absence of an epidemiological link is consistent with more than 12 SNPs, while pairs of 6–12 SNPs were considered to be indeterminate [24].

In our investigation, isolates from the two epidemiologically linked sisters differed by 12 SNPs. This strongly suggests one or more missing links in the transmission chain, namely a common source case for both sisters with possibly additional intermediate cases. Missing links may be the result of undetected TB cases, the restriction of our investigation to only one district in Romania, unavailable genotyping results, or from selection based on identical MIRU-VNTR patterns when a mutation affected a VNTR locus even though isolates differed only by few SNPs [6].

We investigated a single scenario and may not draw conclusions about the extent of cross-border transmission of MDR-TB in the EU. The ECDC MDR-TB molecular surveillance project investigated 2,092 MIRU-VNTR patterns of isolates from 24 contributing EU Member States from 2003 to 2011 [19]. In total, 941 cases in 79 European multiple-country clusters were detected and 1,086 cases were allocated to national clusters. That study was solely based on genotyping data. In the UK, 24-loci MIRU-VNTR typing and epidemiological surveillance data were linked and jointly interpreted, and 8.5% of the MDR-TB cases were attributed to recent domestic transmission [36]. Similar nationwide evaluations are missing for our countries.

A high proportion of imported MDR-TB in low-incidence countries does not necessarily entail ongoing MDR-TB transmission when early case detection, infection control and adequate treatment succeed [19]. A systematic review for the EU/European Economic Area indicates that TB in the foreign-born population has no significant influence on TB in the native population [37].

Beyond higher resolution in TB outbreak investigation, WGS provided us in addition with information on drug resistance of the bacteria. We could identify mutations mediating pyrazinamide resistance in previously not tested isolates and mutations mediating ethambutol resistance in two samples with susceptible phenotypic DST results. However, our data on mutations mediating drug resistance to ethionamide, protionamide and the quinolones showed discrepancies between phenotypical and genotypical DST. A comprehensive database of characterised mutations is needed to extend the usability of WGS in predicting drug resistance, e.g. in order to provide rapid and effective treatment in outbreaks of drug-resistant TB. The concordance of resistance-mediating mutations in each WGS cluster confirmed transmission of MDR strains rather than treatment failure and new acquisition of MDR in each patient [10].

Our investigation was subject to limitations. The collection of direct epidemiological links yielded little information. It was difficult to differentiate whether a specific contact was absent (e.g. due to missing links), unknown (exposures in public space, recall bias) or non-reported (reluctance to name persons). Spatio-temporal data did not cover all patients’ presumed infectious periods and travel history. Their low resolution (per city/country and month) allowed us to judge whether a contact was possible at all, but not to explore new exposure settings or events. More detailed investigations are difficult given long infectious periods and serial intervals in TB transmission chains.

The clinical characteristics ‘cavitary disease’ and ‘HIV status’ were not assessed as they are not notifiable everywhere, although relevant to assessing infectiousness and transmission risks. For patient I, it remained unclear which local public health authority was in charge. This highlights the challenge in transferring patient reports when patients are highly mobile.

We learned that the choice of methods and the order in which we use them can play a significant role. If WGS had been used initially and had led to the detection of the close genetic relationship between isolates from patients II, IV and V in Austria, a cross-border investigation might not have been initiated.

The cross-border investigation of a single genotyping cluster of TB can become complex and labour-intensive with uncertain public health benefits. In our case, there were no implications for contact tracing, which had already been completed. However, such investigation as ours may detect previously undetected individuals with TB. While investigations might get more efficient with increasing routine, each cluster brings together a new group of competent authorities that need to establish collaboration. Systematic and timely integration of genotyping and sequencing data into TB surveillance improves the understanding of transmission in a given country and internationally [38].

Topical issues remain: Should WGS replace 24-loci MIRU-VNTR as a standard? By when? How should we collect, analyse and interpret sequencing data within routine TB surveillance [39] and evaluate utility? How should we prioritise cluster investigations? Are there reliable predictors of cluster growth [40-43]? Will epidemiological links remain an essential component in TB outbreak definitions, i.e. may we use the term ‘outbreak’ solely based on WGS results when epidemiological links cannot confirmed? How can we collaborate most efficiently across borders when contact networks are complex and personal data are to be shared by everyone with everyone else? Could a secure interactive online platform complement communication channels such as EWRS?

## Conclusion

Our joint cross-border investigation clarified a transboundary MDR-TB transmission scenario. The applied methods complemented each other: genotyping results prompted our investigation, classic epidemiological data anchored the cluster in time and space, and WGS allowed a high resolution of transmission and new information on drug-resistance.

To prevent further MDR-TB transmission within and between countries, we need to ensure universal access to early and adequate therapy in order to reduce incentives to seek medical care abroad and to ensure infection control and seamless collaboration in TB care beyond administrative borders [44].
